# CRS: a circadian rhythm score model for predicting prognosis and treatment response in cancer patients

**DOI:** 10.1186/s12967-023-04013-w

**Published:** 2023-03-09

**Authors:** Yuwei Liu, Shuang Guo, Yue Sun, Caiyu Zhang, Jing Gan, Shangwei Ning, Junwei Wang

**Affiliations:** 1 College of Bioinformatics Science and Technology, Harbin Medical University, Harbin, Heilongjiang China; 2 Department of Respiratory Medicine, The Second Affiliated Hospital of Harbin Medical University, Harbin, 150081 China

**Keywords:** Circadian rhythm gene, Prognosis, Immune cell infiltration, Chemotherapy, Immunotherapy, Clock-drugs

## Abstract

**Background:**

Circadian rhythm regulates complex physiological activities in organisms. A strong link between circadian dysfunction and cancer has been identified. However, the factors of dysregulation and functional significance of circadian rhythm genes in cancer have received little attention.

**Methods:**

In 18 cancer types from The Cancer Genome Atlas (TCGA), the differential expression and genetic variation of 48 circadian rhythm genes (CRGs) were examined. The circadian rhythm score (CRS) model was created using the ssGSEA method, and patients were divided into high and low groups based on the CRS. The Kaplan–Meier curve was created to assess the patient survival rate. Cibersort and estimate methods were used to identify the infiltration characteristics of immune cells between different CRS subgroups. Gene Expression Omnibus (GEO) dataset is used as verification queue and model stability evaluation queue. The CRS model's ability to predict chemotherapy and immunotherapy was assessed. Wilcoxon rank-sum test was used to compare the differences of CRS among different patients. We use CRS to identify potential "clock-drugs" by the connective map method.

**Results:**

Transcriptomic and genomic analyses of 48 CRGs revealed that most core clock genes are up-regulated, while clock control genes are down-regulated. Furthermore, we show that copy number variation may affect CRGs aberrations. Based on CRS, patients can be classified into two groups with significant differences in survival and immune cell infiltration. Further studies showed that patients with low CRS were more sensitive to chemotherapy and immunotherapy. Additionally, we identified 10 compounds (e.g. flubendazole, MLN-4924, ingenol) that are positively associated with CRS, and have the potential to modulate circadian rhythms.

**Conclusions:**

CRS can be utilized as a clinical indicator to predict patient prognosis and responsiveness to therapy, and identify potential "clock-drugs".

**Supplementary Information:**

The online version contains supplementary material available at 10.1186/s12967-023-04013-w.

## Introduction

Circadian rhythm is a 24-h endogenous rhythm that controls complex physiological activities in living organisms [1, 2]. In mammals, the circadian rhythm has corresponded to a hierarchical system in which a brain clock located in the hypothalamic suprachiasmatic nucleus (SCN) acts as a master pacemaker to synchronize or entrain the peripheral clocks distributed throughout the body (e.g., liver, kidney, and heart) [3–5]. Circadian dysregulation affects cancer development, including cell cycle control, apoptosis, metabolic regulation, and DNA damage response [6–12]. For example, CLOCK promotes high-grade glioma proliferation and migration [13]. ARNTL induces cell cycle arrest in tumor cells by affecting p53 function [14] and represses tumor cell invasion through PI3K signaling pathway [15]. In addition, recent studies on using circadian rhythm signatures to predict survival and immunotherapy effects in specific tumor types [16–18], as well as studies on the association between circadian clock gene expression and tumor immunity and patient prognosis in specific tumors [19–21]. However, at the pan-cancer level, the study using CRGs to predict the prognosis of patients and therapeutic effects has not been extensively studied, and needs further exploration and verification.

Circadian rhythm is generated and mediated by three autoregulatory transcriptional–translational feedback loops [2, 22]. Genes engaged in circadian rhythm can be divided into two types, the core clock genes and the clock control genes [1, 2, 23]. The former influences the circadian rhythm and is mainly located in the first loop, where CLOCK and BMAL1 form a heterodimer that binds to the E-box elements [24] on the promoters of PER and CRY genes and activates their expression. In turn, PER and CRY proteins accumulate to a critical level and form complexes with CLOCK/BMAL1 or NPAS/BMAL1 heterodimers and thereby repress the transcription of their genes [25, 26]. The latter regulates the expression of core clock genes and is mainly in the second and third loops. Second loop: NR1Ds (REV-ERBs) and RORs, two retinoic acid-related orphan nuclear receptors constitute the stabilizing loop by competing to bind to ROR elements (RRE) [27] in BMAL1. While RORs activate the transcription of BMAL1, REV-ERBs repress the same transcription process [28, 29]. And third loop: CLOCK: BMAL1 drives a loop containing transcription factors such as DBP, TEF, and HLF, which interact with sites containing D-boxes [30] with the repressor NFIL3 driven by the REV-ERB/ROR loop. NFIL3 in turn represses DBP to regulate a rhythm in the RORA/RORB/RORC nuclear receptors [31, 32]. However, research on these two types of CRGs at the pan-cancer level is still in its infancy.

A new strategy for treating cancer involves using drugs that directly or indirectly target the elements of the circadian clock. Two types of clock-drugs are currently available: drugs that can directly modulate the activity of circadian core genes and drugs targeting regulators of circadian core clock genes. Drugs in the second category target proteins that phosphorylate or destroy clock components among other target proteins and thus are not specific for clock components [33]. “Clock-drugs”, such as ROR, REV-ERBs, and CRYs, have recently been created as tools to specifically target circadian clock components [34–36]. For example, RORγ antagonists block the growth of tumors expressing the androgen receptor and restore sensitivity to enzalutamide in resistant tumors [34]. However, "Clock-Drugs" therapy has not yet become the standard practice of cancer treatment, and it has great research potential in the future.

In this study, we first analyzed the overall expression, mutation, and copy number variation of 48 CRGs. We found that most of the core clock genes were up-regulated while the clock control genes were down-regulated. Further analysis found copy number variation as a potential factor affecting CRGs dysregulation. Then, we constructed a circadian rhythm score (CRS) model. We found that the CRS model could distinguish between patients in terms of survival and immunological characteristics. The CRS-low patients had better survival outcomes and lower immune score. We also found that CRS can predict the effect of chemotherapy and immunotherapy in patients with certain cancers. Furthermore, we identified 10 potential "clock-drugs" through CRS that can be used to adjuvant therapy for cancer. In summary, the CRS can be used as a clinical indicator to predict patient prognosis and therapeutic efficacy, and it can also be utilized to identify potential "clock drugs" for cancer adjuvant therapy.

## Methods and materials

### Datasets and source

A total of 48 CRGs, including 13 clock control genes and 35 core clock genes, were obtained when we used the keywords ((((circadian clock gene) OR (circadian rhythm gene) OR (clock control gene) OR (core clock gene)) AND (cancer)) in PubMed (https://pubmed.ncbi.nlm.nih.gov/) (Additional file 4: Table S1).

In the Cancer Genome Atlas data portal (TCGA, https://portal.gdc.cancer.gov/), 18 cancer types (Additional file 4: Table S2) were filtered based on the presence of at least 5 normal samples. We downloaded RNA-Seq data expressed as fragments per kilobase per million (FPKM) from the UCSC Xena browser (GDC Center, https://gdc.xenahubs.net). The gene-expression profiles of TCGA in the Fragments Per Kilobase per Million (FPKM) format were transformed into TPMs (transcripts per kilobase million) by using R version 4.2.0 software. Additionally downloaded for analysis were clinical data, copy number variation data, and single nucleotide variation data.

Microarray datasets including gene expression profiles and corresponding clinical information data of GSE42568, GSE14333, and GSE31908 were employed from the Gene Expression Omnibus database (GEO, https://www.ncbi.nlm.nih.gov/geo/). We downloaded the chemotherapy and immunotherapy response datasets from the GEO database (GSE25055, GSE20194, GSE42127, GSE14814, GSE146163, GSE78220, and GSE174570). Moreover, we acquired four sets of mice's expression profile data from the GEO database that had circadian rhythm genes knocked out (GSE134333, GSE188688, GSE134333, and GSE143524). For microarray data, we performed log2 conversion of the normalized expression profile data. The list of these datasets was displayed in Additional file 4: Table S3.

### Identifying differences in circadian rhythm genes expression

Gene expression profiles were collected from the TCGA project to examine genes that are differently expressed between tumor and normal tissue. Then, the fold change and adjusted P-value were calculated by the 'limma' package [37]. We defined genes with P values less than 0.05 and absolute values of fold change greater than 2 as differentially expressed genes (DEGs).

### Analyzing the features of single nucleotide variation and copy number variation

Single nucleotide variation (SNV) data (n = 7130) were collected across 18 cancer types from Genomic Data Commons. We estimated the mutation frequency of each circadian rhythm gene in each tumor using the maftools (https://bioconductor.org/packages/release/bioc/html/maftools.html) and oncoplot waterfall plot [38]. We show genes with an overall mutation proportion of over 10%. SNV mutation frequency (percentage) of each gene's coding region was calculated using the formula: Number of Mutated Samples/Number of Cancer Samples. The SNVs were divided into missense mutations, nonsense mutations, multiple hits, frameshift insertions, frameshift deletions, splice sites, in-frame amplifications and in-frame deletions.

For copy number variation (CNV) data, we downloaded gene level copy number score data (GISTIC—focal score by gene) from UCSC Xena, which is the sequence interval focused on the gene and assessed whether the gene was amplified or deleted. Values between − 0.3 and 0.3 were scored as 0 for no changes, larger than 0.3 as 1 for amplifications, and less than 0.3 as − 1 for deletions. Heatmap plot of mutation frequency was generated by using ComplexHeatmap R package.

### Constructing the circadian rhythm score model

The score to represent the circadian rhythm disorder level was established based on the expression data of CRGs, including 13 clock control genes and 35 core clock genes. The single sample gene set enrichment analysis (ssGSEA) [39] in the R package "GSVA" was used to find equally enriched pathways and calculate the gene set enrichment scores (ES) for clock control genes and core clock genes. The control component minus the core component was defined as the circadian rhythm score (CRS) to quantify the expression levels of these genes for each cancer patient. We also estimated the CRS between tumor and normal samples in 18 cancers from the TCGA. We use the surv_cutpoint function of the "survminer" R package to determine the optimal cut-point for CRS and to divide patients into high and low subgroups.

### Survival analysis

We compared the overall survival (OS) of cancer patients separated by the best cut-point of CRS in each cancer type. Kaplan–Meier (K–M) curves were used to compare the differences in survival times. P-values from log-rank tests were computed, and statistical significance was defined as less than 0.05.

### Investigating variations in immune cell infiltration

The gene expression data from the TCGA cohorts was examined using the CIBERSORT program (http://cibersort.stanford.edu/) to produce a fraction matrix of CRS groups, which calculates the abundances of 22 different leukocyte subsets [40]. The mRNA expression matrix was imported into CIBERSORT and iterated 1000 times to assess the proportion of immune cell in each cancer sample of patients. Variations in the level of immune cell infiltration in the two CRS groups were measured with the help of the Wilcoxon rank-sum test (p values < 0.05). Based on the proportion of immune cell infiltration and the expression of CRGs in each sample, we used spearman correlation analysis to determine the link between the type of immune microenvironment infiltrating cells and CRS for each tumor.

Using the gene expression data, we implemented the Estimate algorithm [41] to predict the presence of stromal and immune cells in malignant tumor tissues. We assessed the differences between groups based on a t-test focused on the stromal score, immune score, and tumor purity information of different groups.

### Correlation analysis of immune checkpoint inhibitors

In total, 60 immune checkpoint genes (ICGs) were identified from the literature [42–45], and most of the ICGs were ligands, receptors, or important molecules in immune checkpoint pathways (Additional file 4: Table S4). We computed the Spearman's correlation between CRS and immune checkpoint genes (PD-1, PD-L1, and CTLA-4) to examine the association between CRS and the expression patterns of ICGs.

### Identifying potential “Clock-Drugs”

The Connective Map (CMap) method is a gene expression data-driven approach for uncovering links among genes and chemicals [46, 47]. The CMap library is a collection of gene expression profiles from human cancer cells that have received pharmacological treatment. CMap collects more than 7000 gene expression profiles representing 1309 compounds. Since most CMap compounds are FDA-approved drugs, the database has become a powerful tool for drug repurposing. Here, we use the Connection map online tool (http://clue.io) to perform a connectivity map analysis of the top 150 positive and negative linked genes for CRS for each cancer type. Significant positive or negative compounds were those with enrichment scores of more than 90 or less than -90 in at least 10 cancer types, respectively.

## Results

### Two types of CRGs have different transcriptomic characteristics in cancers

**Fig. 1:**
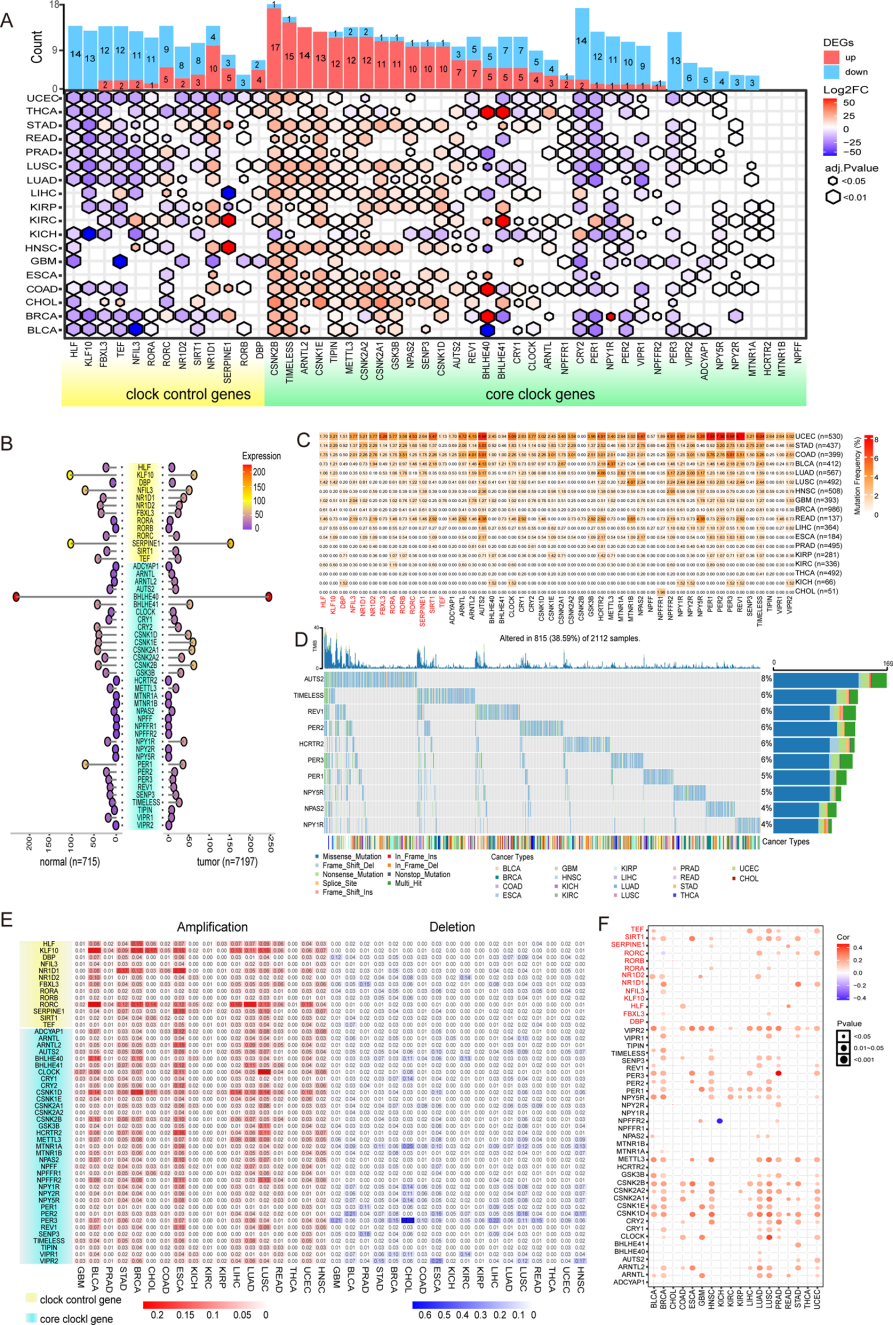
Abnormal patterns in expression of circadian rhythm genes in Cancer. **A** The mRNA difference between normal samples and tumor samples. Only significant differential expressed genes were shown. At the top of the figure is the summary of mRNA alteration of CRGs. **B** Average CRGs expression in cancer. **C** Mutation frequency of CRGs. **D** Mutation type and summary of CRGs in cancers. **E** Copy number variation amplification/deletion of CRGs in cancers. **F** Copy number variation associates with gene expression of CRGs

13 clock control genes and 35 core clock genes were collected from the earlier studies. These 48 genes' protein–protein interaction network (Additional file 1: Fig. S1A) reveals the intricate control of circadian rhythm. We retrieved the transcriptome and genome profile of cancers from the Cancer Genome Atlas. Based on the gene expression data from TCGA, we implemented "limma" to calculate the difference of CRGs for 18 different cancer types in order to compare the CRGs between tumor and normal samples. These two types CRGs significantly differed in cancers. Among 7912 samples across 18 cancer types, we interestingly observed that the majority of the clock control gene, such as HLF, KLF10, FBXL3, TEF, RORs (RORA, RORB, RORC), and NR1D2, are down-regulated in a wide range of cancers (Fig. 1A). However, most of core clock genes (CSNK2B, TIMELESS, ARNTL2, CSNK1E, TIPN, METTL3, etc.) are up-regulated or unchanged (HCRTR2, MTNR1B, and NPFF). This outcome suggests that the circadian rhythm loses its regular daily rhythmic activity cycle in cancer [43].

Then, we illustrated the average expression of CRGs in 7197 tumor samples and 715 normal samples using a lollipop plot (Fig. 1B) to explore the variations in CRGs expression. Among the clock control genes that tend to be down-regulated, the gene SERPINE was observed to be more expressed in tumors than in normal samples, and higher than genes within the same type. In Fig. 1A also shows that this gene exhibits an up-regulated pattern in most cancers. It has been reported that SERPINE1 and ARNTL2 expression is increased in colorectal cancer and in a highly proliferative colon cancer cell line and is related to tumor invasiveness and aggressiveness [48]. The BHLHE40 gene was also considerably more expressed in normal and tumor samples than any other core clock gene. In Fig. 1A a consistent proportion of the number of this gene was found to be up-and down-regulated in tumors. The expression patterns of BHLHE40 and its impact on tumor development are tumor type-specific it is suppressed in some types of cancer and overexpressed in others. The basic-helix-loop-helix (BHLH) protein BHLHE40, which is overexpressed in gastric, breast, and brain tumors; and downregulated in colorectal, esophageal, pancreatic, and lung cancer [49].

To further understand why CRGs are transcriptionally dysregulated, we analyze SNV and the CNV in 18 cancer types. We first examined genomic profiles and found that most CRGs were mutated at low frequencies (Fig. 1C, Additional file 4: Table S5). The CRG frequencies of UCEC, STAD, and COAD were 2–8% in these cancers. In 2112 analyzed patients, 815 (38.59%) show at least one mutation (Fig. 1D). Missense mutation is a major variant event. The genes AUTS2, TIMELESS, PER3, PER2, and REV1 are with relatively high mutation frequency among 48 CRGs. Our results suggest that SNV may not be a factor in the abnormal circadian rhythm of cancer. This is consistent with the findings of other studies [50]. Next, we examined CNV in 18 cancer types with very low frequencies of copy number deletions and amplifications. Individual CRGs displayed cancer type dependent amplification or deletion pattern. RORC was found to have a high amplification frequency in BLCA (22.4%), and LUAD (20.5%). KLF10 amplification predominately occurred in BLCA (20.9%), BRCA (18.4%), and CHOL (16.7%) (Fig. 1E, Additional file 4: Table S6, S7). In addition, the loss of PER3 was significantly found in CHOL (69.4%), GBM (20.7%), and LIHC (21.6%). Our findings indicate that transcriptional dysregulation of some CRGs is likely the root cause of copy number amplification or deletion. To further support our conclusion, we perform a correlation analysis between CNV and mRNA level. As is seen in Fig. 1F, in the majority of cancer tissues, the CNV is associated with the mRNA level [43]. We analyzed the difference of gene expression between normal and cancer samples, and found that the deletion of PER3 in CHOL significantly reduced the expression level (Additional file 1: Fig. S1B). As a result, different types of CRGs have varied transcriptomic and genomic characteristics in cancer, and CRGs dysregulation is linked to copy number alteration.

### Construction of circadian rhythm score model to recognize patient groups with specific prognostic features

**Fig. 2:**
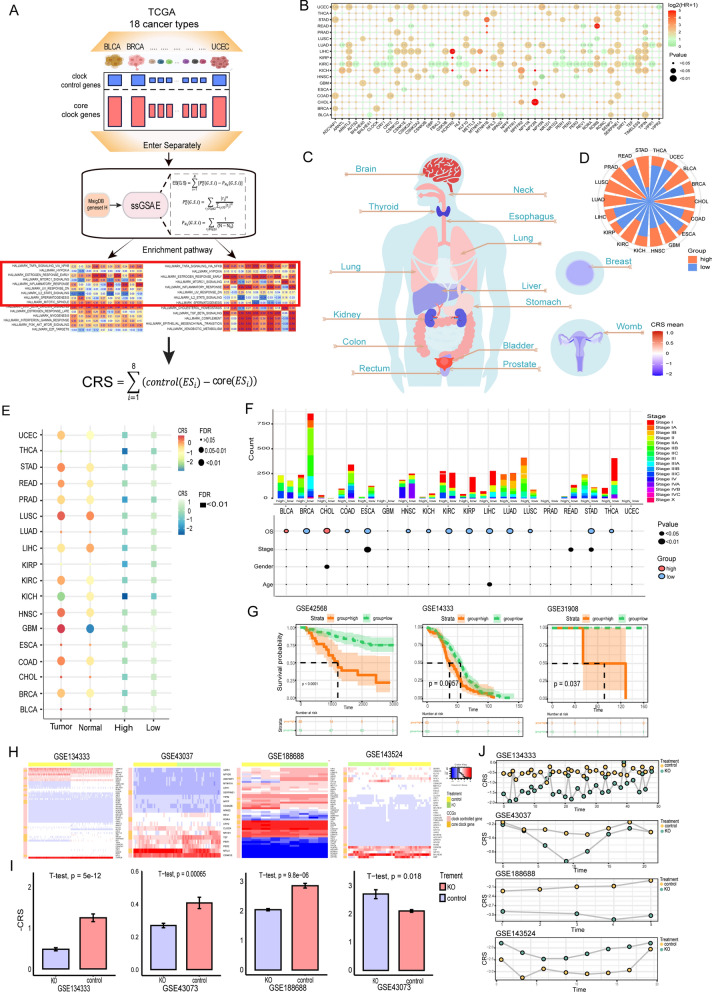
Construction and analysis of the circadian rhythm score model. **A** Flow chart of CRS model construction. **B** Univariate cox regression analysis of CRGs in cancer. **C** Distribution of CRS values in organs. **D** Percentage of samples with CRS-high and CRS-low classifications across 18 cancer types based on the optimal threshold of CRS as the cutoff. **E** Difference of CRS between tumor samples and adjacent normal samples and CRS subgroups across 18 cancer types. **F** Differences in survival time and clinical characteristics among CRS subgroups. **G** Survival analysis of GEO validation data. **H** Heat map of CRGs expression. **I** Bar-compartmental plots of CRS in experimental and control groups. **J** The rhythm of CRS over time in four validated datasets

To further understand the role of circadian rhythm in tumorigenesis and investigate the functional implications associated with CRGs, the circadian rhythm score (CRS) model was based on the enrichment score (ES) of clock control components calculated by ssGSEA minus that of core clock components (Fig. 2A). In order to determine the significance of CRS in predicting patient prognosis, we first did a univariate cox regression analysis of CRGs (Fig. 2B), and we found that, with the exception of KIRC, the majority of CRGs were present as risk factors in malignancies. For instance, the SERPINE1 gene was present as a risk factor in eight cancer types. Univariate Cox regression analysis identified CRGs as statistically significant clinical factors associated with overall survival (OS). For all samples in the TCGA cohort, we estimated CRS values, and for tumor patients, we evaluated CRS distribution (Fig. 2C). Unexpectedly, we found that CRS had significant organ selectivity, with tumors from the brain and digestive system typically having higher CRS values, while tumors from some urinary systems and some secreting glands, such as the liver, kidney, prostate, thyroid, and breast invasive carcinoma, which typically have lower scores.

To analyze the prognostic value of CRS, we divided the tumor samples into high and low CRS groups according to the optimal threshold (Fig. 2D). In the beginning, we used T-tests to assess CRS values between normal and tumor samples and CRS subgroups. We observed significant differences between normal and tumor CRS values in most (12/18) cancer types, where higher CRS was noticed in tumor patients (Fig. 2E, Additional file 1: Fig. S1C). Using Kaplan–Meier survival analysis, we evaluated OS between various CRS groups (Fig. 2F, Additional file 2: Fig. S2A), and we found that patients with CRS-high had significantly lower OS than patients with CRS-low in 12 cancer types. Additionally, we gathered and compiled clinical characteristics like patient age, stage, and gender (Fig. 2F, Additional file 2: Fig. S3A) to investigate the variations in clinical factors of patients between CRS subgroups. The analysis revealed no appreciable variations in clinical features between patients in CRS subgroups. To further research the correlation between the expression levels of CRGs in cancer and the clinical features of patients, we used a chi-square test to examine the relationship between CRGs and patients' clinical factors (age and gender) (Additional file 2: Fig. S2A, B). The finding showed that 22 CRGs in KRIP and 15 CRGs in BRCA were strongly connected with age (P < 0.05), while 22 CRGs in KIRC were significantly correlated with gender (P < 0.05). We also analyzed the relationship between CRS and patient age and gender (Additional file 2: Fig. S2C), and we found that CRS was not statistically significant with age and gender in most cancers. In conclusion, the complexity of cancer makes the association of differential expression of CRGs with age and sex insignificant.

To further evaluate the prognostic value of CRS, we performed validation using three GEO cohorts (n = 429, Fig. 2G), and the results showed that patients with low CRS subgroups had better survival in primary lung cancer (GSE31908), primary colorectal cancer (GSE14333), and breast cancer (GSE42568). All of these findings suggest that CRS is a valuable prognostic predictor and the potential predictive power of our CRS model classification. To assess the performance and robustness of CRS, we collected independent gene expression datasets [51–53] involving murine models that were either exposed to circadian deregulation treatment (CDT) or not [54]. While GSE143524 was a cohort with double knockout of clock control gene REV-ERB a/b (NR1D1/2), GSE134333, GSE43037, and GSE188688 were cohorts with knockout of core clock gene Bmal1 (ARNTL) (Fig. 2H). We found that the CRS values following knockout of the core clock gene Bmal1 were much lower than the control group, whereas the CRS values after knockout of the clock control gene REV-ERB a/b were significantly higher than the control group (Fig. 2J, vertical coordinate: -CRS). Plotting the CRS fluctuations over time for samples using CDT and samples under normal settings revealed that the CRS fluctuations were lower for samples using CDT than for samples under normal circumstances (Fig. 2I).

### The different CRS groups possess distinctive immune cell infiltration traits

**Fig. 3:**
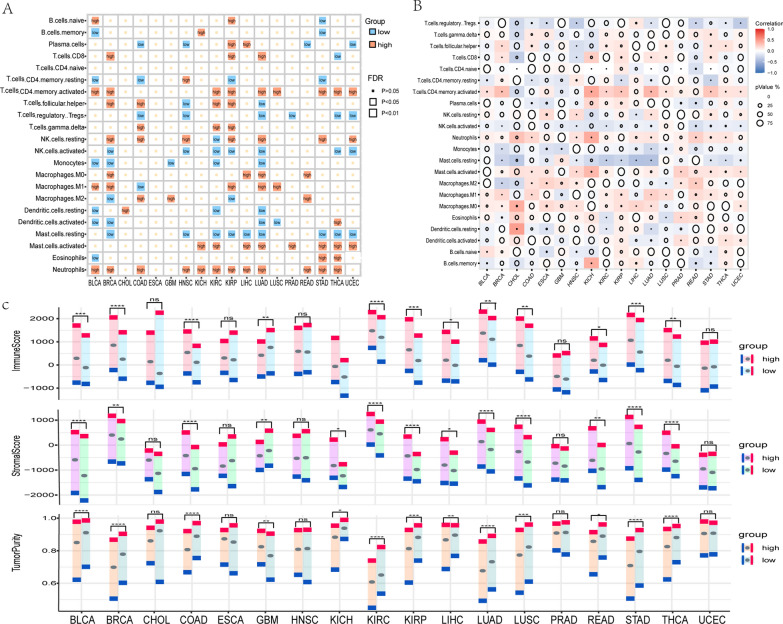
Immune infiltration characterization of CRS subgroups. **A** Differences in immune cell infiltration between high and low CRS subgroups. **B** Heat map of the correlation between the percentage of immune cell infiltration and CRS. **C** Differences in immune score, stromal score and tumor purity between CRS subgroups

The immune system is under the control of the circadian clock. The main means of circadian control over the immune system is by direct control of circadian clock proteins acting as transcription factors driving the expression or repression of immune genes [55]. In order to reveal the immune cell infiltration characteristics in the immune microenvironment of various CRS subgroups, we computed 22 types of immune cell infiltration in different subgroups using the Cibersort algorithm (Fig. 3A). The findings showed that different cancer types had different proportions of immune cell infiltration due to the heterogeneity of cancer. For instance, there were 12 immune cell types with infiltration proportions in BRCA while there were 9 immune cell types with infiltration proportions in COAD. However, the features of immune cell infiltration were similar across cancer types within the same CRS group. For example, the majority of infiltrating immune cells in the CRS-high group included CD8 T cells, activated CD4 memory T cells, gamma T cells, resting NK cells, activated mast cells, neutrophils, and CD4 memory T cells, which are mostly involved in anti-tumor and inflammatory response processes. We also calculated the correlation between the abundance of tumor-infiltrating immune cells and CRS in 18 cancer types (Fig. 3B, Additional file 4: Table S8). We observed that the majority of the infiltrated cell types in the CRS-high group had a positive connection with CRS, whereas most of the infiltrated cell types in the CRS-low group showed a negative correlation with CRS. This correlation ratio is also linked with cancer heterogeneity. For example, there were 7 and 17 types of tumors positively connected with CRS in CD8 T cells and neutrophils, respectively, while the differences in cancer infiltration in the CRS-high group were 3 and 12 types, respectively.

To further demonstrate the differences in immune cell infiltration characteristics among CRS subgroups, we analyzed the immune cell scores of CRS subgroups by applying the ESTIMATE method to 7197 samples from 18 cancer types (Fig. 3C). The result showed that immune cell infiltration varied significantly across 13 cancer types, and that tumor purity was significantly higher in the CRS-low group. In addition, the immune score and stromal cell score were significantly lower in the CRS-low group, with the exception of glioma (GBM). In general, the CRS-high group had a higher immune score and had more immune cells engaged in inflammatory and anti-tumor responses.

### The CRS can predict the chemotherapy response of patients

**Fig. 4:**
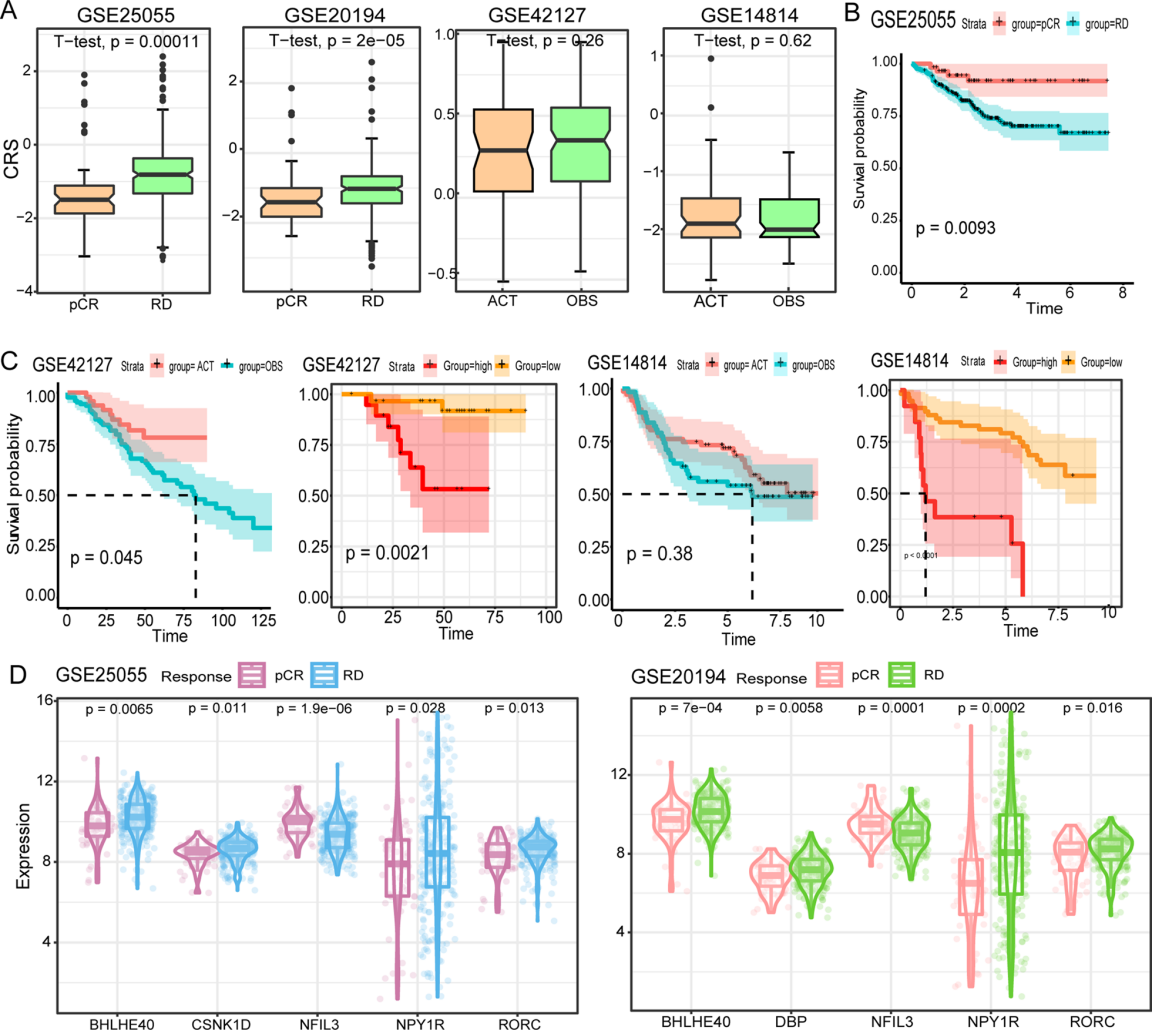
CRS predictive chemotherapy response analysis. **A** Comparative compartmental plots of CRS values for different subgroups. Breast cancer cohort (GSE25055, GSE20194): complete and partial response (PCR), no response (RD); non-small cell lung cancer cohort (GSE42127, GSE14814)): receiving chemotherapy (ACT), control (OBS). **B** GSE25055 subgroup survival analysis. **C** Survival analysis of different subgroups of non-small cell lung cancer. **D** Violin plots of differential CRGs expression in GSE25055 and GSE20194 subgroups

Since the CRG was closely linked to the prognosis of patients, we then tested whether the CRS could accurately predict clinical outcomes. In four separate BRCA and LUAD groups that received chemotherapy, we investigated at CRS's capacity to predict the treatment's outcomes. Patients who responded to chemotherapy in the breast cancer cohort (GSE25055, GSE20194) had much lower CRS values than non-responders (Fig. 4A), and they had significantly better treatment response (Fig. 4B). In contrast, we observed no significant difference in CRS values or survival time between chemotherapy patients (ACT) and controls (OBS) in the non-small-cell lung cancer cohort (GSE42127, GSE14814) (Fig. 4C). After that, we divided up the patient getting chemotherapy into two groups. Surprisingly, we found that the CRS-low patients had a more significant treatment effect, and had a higher 5-year survival rate than the CRS-high patients (Fig. 4C). To investigate which CRGs were significantly altered in responding patients, we analyzed the BRCA cohort patients (Fig. 4D). The results showed that the expression of up-regulation core clock genes (BHLHE40, CSNK1D) was significantly reduced, while the expression of down-regulation clock control genes (NFIL3, DBP, RORC) was significantly increased. These findings imply that CRS has the capacity to predict the response of chemotherapy.

### The CRS has the capability to evaluate patients' reactions to immunotherapy

**Fig. 5:**
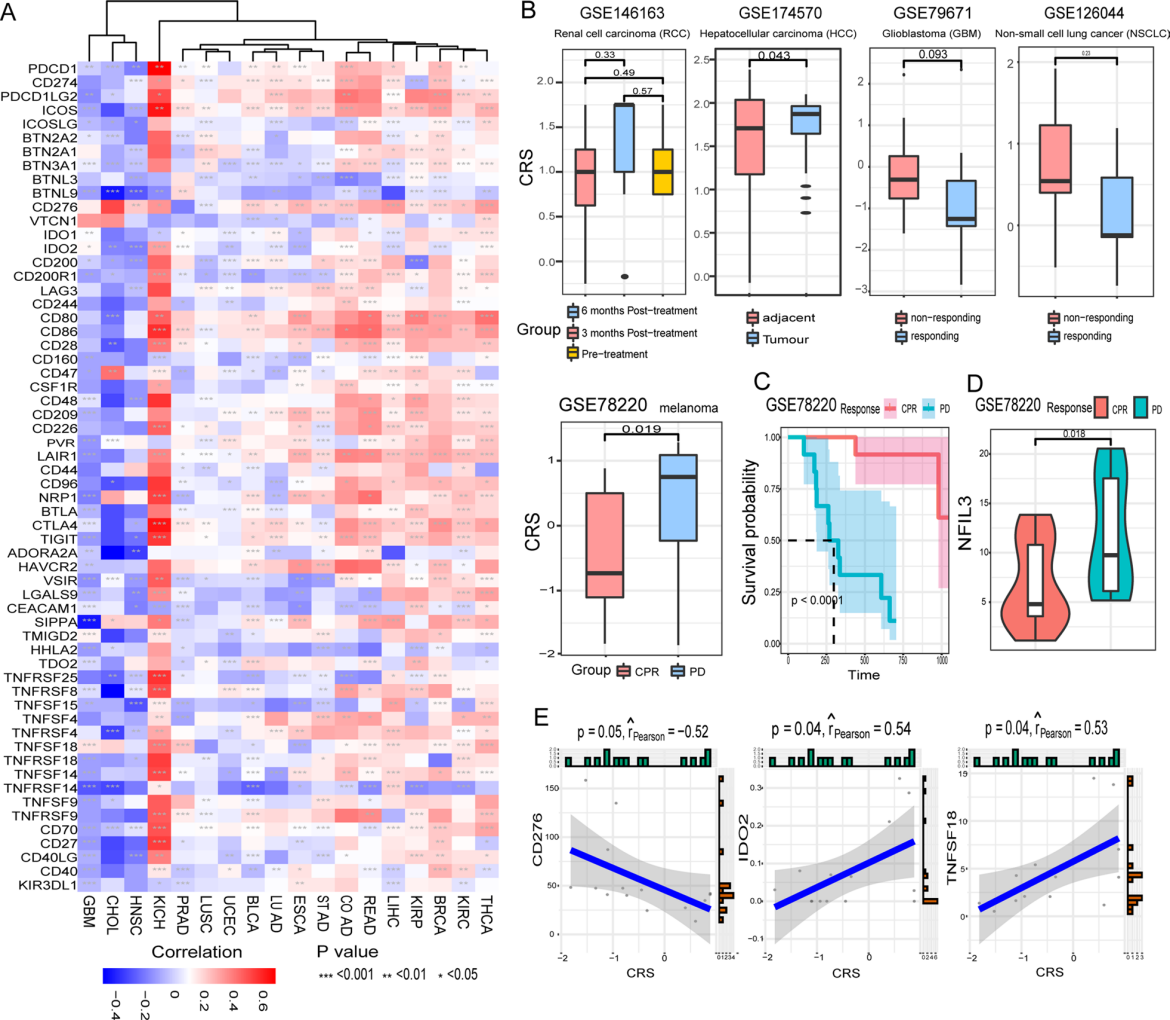
CRS assessment of immunotherapy response analysis. **A** Heat map of the correlation between CRS and ICGs in 18 cancer types. The asterisk character represent the significance of the statistical difference: *p < 0.05; **p < 0.01; ***p < 0.001. **B** Compartmental plots of CRS in patients with different immunotherapy subgroups. **C** Survival analysis of GSE78220 subgroups. **D** Differential expression of NFIL3 gene in GSE78220 subgroups. **E** ICGs significantly associated with CRS in GSE78220

We investigate whether CRS can predict that effect of immunotherapy. Using Pearson correlation analysis, We found four cancer subgroups based on the correlation of 60 ICGs with CRS (Fig. 5A, Additional file 4: Table S9). These included the strong positive group (KICH), the positive group (COAD, READ, and KIRC, etc.), the strong negative group (GBM, CHOL, and HNSC), and the negative group (PRAD, LUAD, and BLCA, etc.). Five immunotherapy data sets for renal cell cancer (GSE146163), hepatocellular carcinoma (GSE174570), glioma (GSE79671), non-small cell lung cancer (GSE126044), and melanoma (GSE778220) were gathered to examine the distinctions between these groups. By comparing various immunotherapy subgroups or stages, we noticed that CRS increased in patients treated in the positive group, and decreased in patients treated or responding to treatment in the negative group (Fig. 5B). Patients with melanoma who responded to treatment had much lower CRS values than those who did not, and those who responded had significantly greater survival rates than those who did not (Fig. 5C). We hypothesized that the lower CRS in responding patients might be associated with a significant reduction in the NFIL3 gene by analyzing and comparing the GSE78220 cohort (Fig. 5D). We further calculated the correlation between CRS and 60 ICGs in GSE78220, and obtained five ICGs that were significantly correlated with CRS, namely CD276, IDO2, TNFRSF4, TNFSF14 and TNFSF18, by screening the correlation coefficient with absolute values greater than 0.7 and P values less than 0.05 (Fig. 5E), among which only CD276 gene showed a significant negative association with CRS. In summary, CRS can be used as an indicator for immunotherapy in the negative group of cancers such as melanoma, glioma, and non-small cell lung cancer.

### The CRS identify potential compounds with circadian regulatory functions

**Fig. 6:**
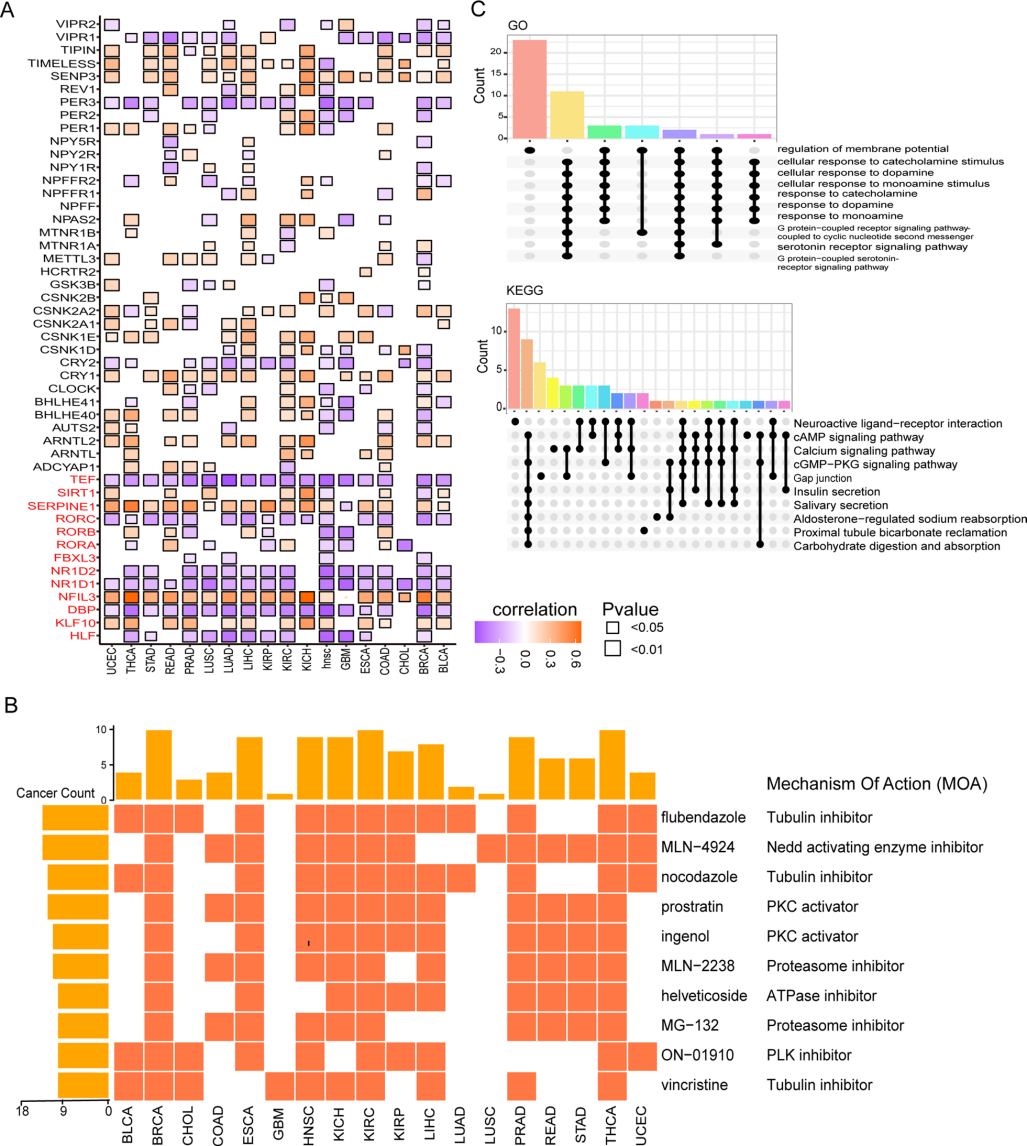
Analysis of compounds potentially regulating circadian rhythms. **A** Heat map of CRS correlation with 48 CRGs in 18 cancer types. **B** Compounds positively correlated with CRS. **C** Target gene enrichment analysis of positively correlated compounds (GO and KEGG)

First, we calculated the Pearson correlation between 48 CRGs and CRS, and we found a significant association between CRS and CRGs that were abnormally upregulated in cancer (Fig. 6A). Together with the above analysis, we came to the conclusion that up-regulated CRGs are mostly to blame for higher CRS values, which have an impact on patient prognosis and therapeutic response. Next, we used the Connective Map method to search for potential compounds that could target circadian rhythm-related pathways or modulate the expression of genes related to circadian rhythm as “clock-drugs”. By setting strict thresholds for association with CRS in at least 10 of 18 cancer types, we found 10 compounds (Fig. 6B, Additional file 4: Table S10) positively correlated and 27 negatively correlated (Additional file 3: Fig. S3B). Among these compounds, six protein inhibitors (including antitumor activity inhibitors, proteasome inhibitors, antiarrhythmic inhibitors, etc.) were found to be positively associated with CRS, implying that positively associated inhibition may regulate CRGs expression. We identified compounds that directly target CRGs, such as cycloheximide, SB-415286, BX-795, GSK-3-inhibitor-II and kenpaullone targeting the GSK3B gene, digoxin targeting the RORC gene, and troglitazone targeting the SERPINE1 gene.

To further analyze the effects of these compounds, we performed functional enrichment analysis of the target genes of the compounds and found that they were mainly enriched for "response to dopamine", "serotonin receptor signaling pathway", "cellular response to monoamine stimulus", "response to monoamine", and "response to catecholamine" and other biological processes (Fig. 6C). These positively correlated compounds primarily modulate neurotransmitters such as dopamine, monoamine, catecholamines, and serotonin to regulate circadian rhythm.

## Discussion

A large body of evidence has indicated that the CRG (CLOCK, BMAL1, PER, CRY, etc.) is associated with tumor progression, prognosis, and treatment response [9, 56–67]. On the other hand, in some cancers, BMAL1 or CLOCK act as oncogenes [13, 68, 69], but in other cancers and model systems, their targeting is tumor suppressive [70–72]. Thus, we can recognize the CRG as a double-edged sword towards cancer, suggesting that it can prevent cancer occurrence or in the completely opposite way promote tumor development in certain types of cancer. Considering the attractive function of the circadian rhythm in tumorigenesis and tumor development, it can be highly significant to analyze the CRG in cancer.

By comprehensively analyzing the genomic and transcriptomic data of 48 CRGs in TCGA, we characterized the expression of CRGs in different cancer types. Heterogeneous distribution of CRGs was observed in cancers: clock control genes such as HLF and RORs were lowly expressed in most tumors, while core clock genes such as TIMELESS and NPAS2 were highly expressed. However, other CRGs, including NR1D1, PER, and CRY, display discrepancies with the results. REV-ERBα (also known as NR1D1) is a nuclear receptor and a core component of the molecular clock system [73]. NR1D1 function as transcriptional repressors, and inhibit gene transcription by recruiting co-repressors nuclear receptor co-repressor 1 (NCOR1) and histone deacetylase 3 (HDAC3) [74]. A large portion of clock-controlled genes are under the control of NR1D1 and show characteristic patterns antiphase to NR1D1 [73]. Period (PER) and cryptochrome (CRY) rhythmically repress the activity of the circadian transcription factor, CLOCK: BMAL1, to establish daily patterns of gene expression [75]. Similar to clock control genes, PERs and CRYs are variably down-regulated in core clock genes. There is evidence that PER1 and CRY1/2 regulate gene expression outside the core regulatory feedback loop [1], which means that PERs and CRYs genes have the function of clock control.

Pan-cancer single nucleotide variation analysis showed that the overall average mutation frequency ranged from 0 to 8.2%, and CRGs including AUTS2, TIMELESS, REV1, and PER2 showed high mutation frequencies. The AUTS2 gene is associated with multiple neurodevelopmental disorders and neurological disorders [76–79]. AUTS2 mutation is linked to autism spectrum disorders [80], epilepsy, cerebral palsy, facial dimorphism [81], developmental delays [82], mental retardation [83], and schizophrenia [84], among other disorders. TIMELESS promotes the progression of lung cancer [85], colorectal cancer [86], ovarian cancer [87], and breast cancer [88] and is associated with patient prognosis. It is worth noting that most CRGs were frequently mutated in UCEC, a certain type of cancer with a high global mutation burden [89]. In addition, the copy number variations investigation revealed that RORC and CSNK1D genes showed widespread copy number amplification across various cancer types whereas almost no CNV was detected in THCA, KIRC, and KIRP.

Univariate cox regression analysis showed that CRGs exhibited a cancer-promoting effect in UCEC, THCA, STAD, LUAD, LIHC, KICH, GBM, COAD, CHOL, and BRCA patients. In contrast, the opposite effect was found in KIRC patients. Prognostic analysis showed that patients in the CRS-high group among 14 cancer types were associated with having poorer survival rates and higher immune scores. These results suggest that the CRS model can differentiate patient prognosis as well as the level of immune cell infiltration. We also looked into the possibility of CRS predicting patient response to immunotherapy and chemotherapy. The findings demonstrated that CRS has predictive value in identifying particular cancer types (breast cancer, non-small cell lung cancer, glioma, and melanoma) and that individuals with low CRS are more responsive to treatment and had higher survival rates. This implies that CRS might be employed as a clinical indicator to forecast treatment outcomes and find potential “clock drug”.

## Conclusion

In summary, our study systematically demonstrated transcriptional and genomic characteristics of two types of circadian rhythm genes. CRS model can predict the prognosis of cancer patients and distinguish the characteristics of immune cell infiltration in patients. We found that CRS may be a potential biomarker of response to chemotherapy and immunotherapy in specific cancers. These findings provide new options for future research on the application of circadian rhythm in cancer development and treatment. In addition, we identified potential "clock drugs" based on CRS that could be used for adjuvant cancer treatment.

## Supplementary Information

**Additional file 1: Figure S1.** Difference of circadian rhythm gene expression and interaction network. **(A)** The protein–protein interaction network of circadian rhythm genes. **(B)** Comparison of expression levels of RORC, CLOCK, PER3 and VIPR2 genes in individual cancer types. **(C)** Comparison of CRS values between cancer and normal by T test. The asterisk character represent the significance of the statistical difference: ns, p > 0.05; *p < 0.05; **p < 0.01; ***p < 0.001.

**Additional file 2: Figure S2.** Correlation of circadian rhythm genes expression levels with clinical features in cancer. **(A)** Correlation of circadian rhythm genes expression levels with age: *p < 0.05; **p < 0.01; ***p < 0.001. **(B)** Correlation of circadian rhythm genes expression levels with gender. **(C)** Correlation of CRS with age (left) and sex (right).

**Additional file 3: Figure S3.** Comparison of CRS group survival and compounds negatively correlated with CRS. **(A)** Comparing the survival time of CRS subgroup samples by Log-rank method. **(B)** 18 compounds negatively correlated with CRS were identified by Cmap analysis.

**Additional file 4: Table S1.** The information of 48 circadian rhythm genes. **Table S2.** The information about the 18 caner types of TCGA cohort. **Table S3.** The information of all GEO cohort, including validation cohort, mouse knockout cohort, and therapy cohort. **Table S4.** The information of immune checkpoint genes. **Table S5.** The mutation frequency of circadian rhythm genes across 18 cancer types. **Table S6.** The CNV amplification frequency of circadian rhythm genes across 18 cancer types. **Table S7.** The CNV deletion frequency of circadian rhythm genes across 18 cancer types. **Table S8.** The correlation of CRS and immunocyte infiltration ratio. **Table S9.** The correlation of CRS and immune checkpoint genes. **Table S10.** The information of compounds positively related to CRS.

## Data Availability

All data used in our study are publicly available. RNA-seq data and corresponding clinicopathological characteristics of 18 cancer types were downloaded from TCGA (https://tcga-data.nci.nih.gov/tcga/). The data of GSE31908, GSE14333, GSE42568, GSE134333, GSE188688, GSE134333, GSE143524, GSE25055, GSE20194, GSE42127, GSE14814, GSE146163, GSE78220, GSE79671, and GSE174570 were obtained from GEO (https://www.ncbi.nlm.nih.gov/geo/).

## Abbreviations

CRG: Circadian rhythm gene
CRS: Circadian rhythm score
SCN: Suprachiasmatic nucleus
TCGA: The Cancer Genome Atlas
GEO: Gene Expression Omnibus
DEG: Differentially expressed genes
SNV: Single nucleotide variation
CNV: Copy number variation
ssGSEA: Single-sample gene set enrichment analysis
GSVA: Gene set variation analysis
OS: Overall survival
ES: Enrichment score
ICG: Immune checkpoint gene
CMap: Connective Map
