# Supplementary figures and images for: CRS: a circadian rhythm score model for predicting prognosis and treatment response in cancer patients
Source: J Transl Med. 2023 Mar 9;21:185. doi: 10.1186/s12967-023-04013-w (PMC9996877; doi:10.1186/s12967-023-04013-w)

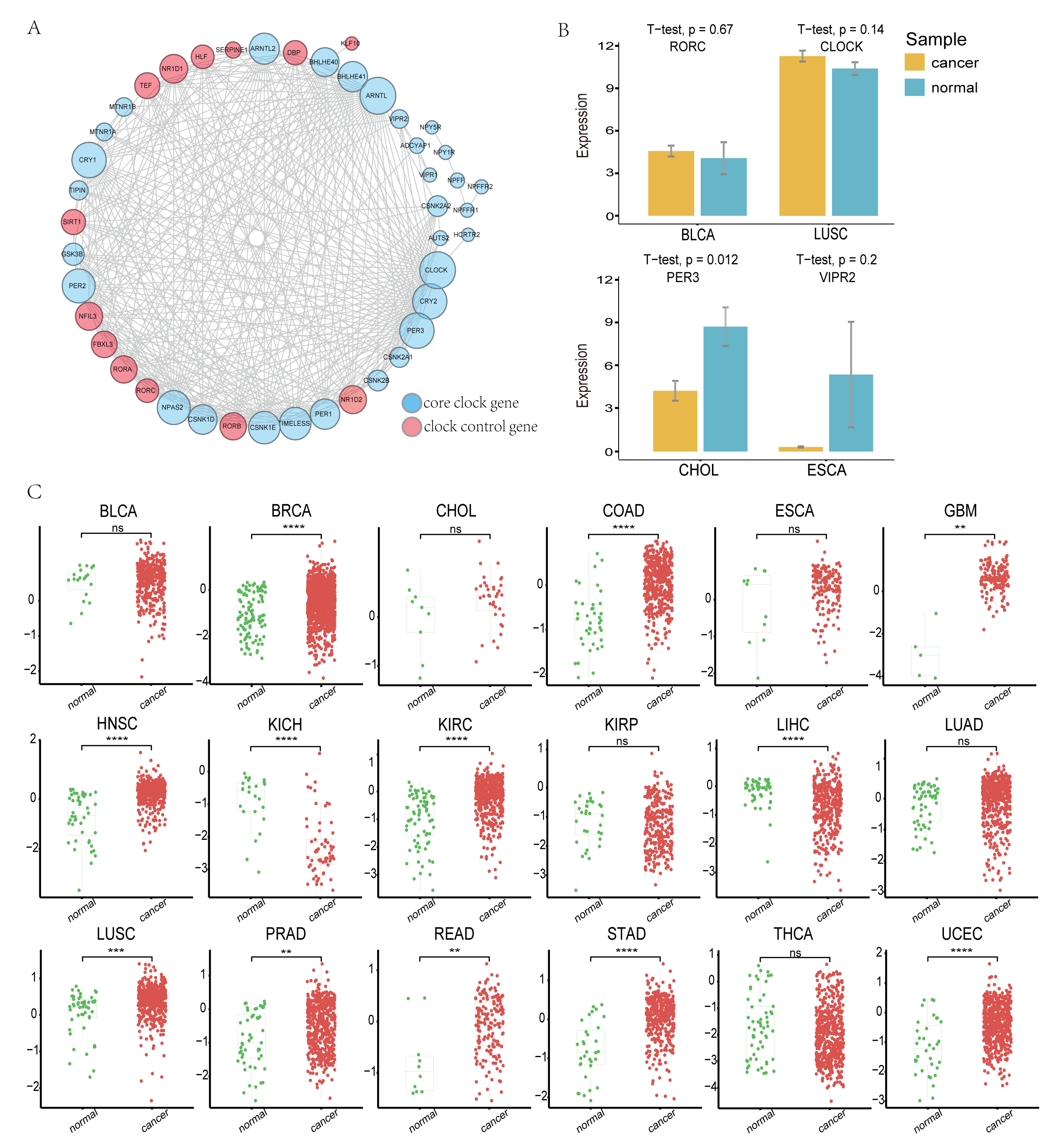

Supplement: Supplementary file 1 — Additional file 1: Figure S1. Difference of circadian rhythm gene expression and interaction network. (A) The protein–protein interaction network of circadian rhythm genes. (B) Comparison of expression levels of RORC, CLOCK, PER3 and VIPR2 genes in individual cancer types. (C) Comparison of CRS values between cancer and normal by T test. The asterisk character represent the significance of the statistical difference: ns, p > 0.05; *p < 0.05; **p < 0.01; ***p < 0.001. [file 12967_2023_4013_MOESM1_ESM.tif]

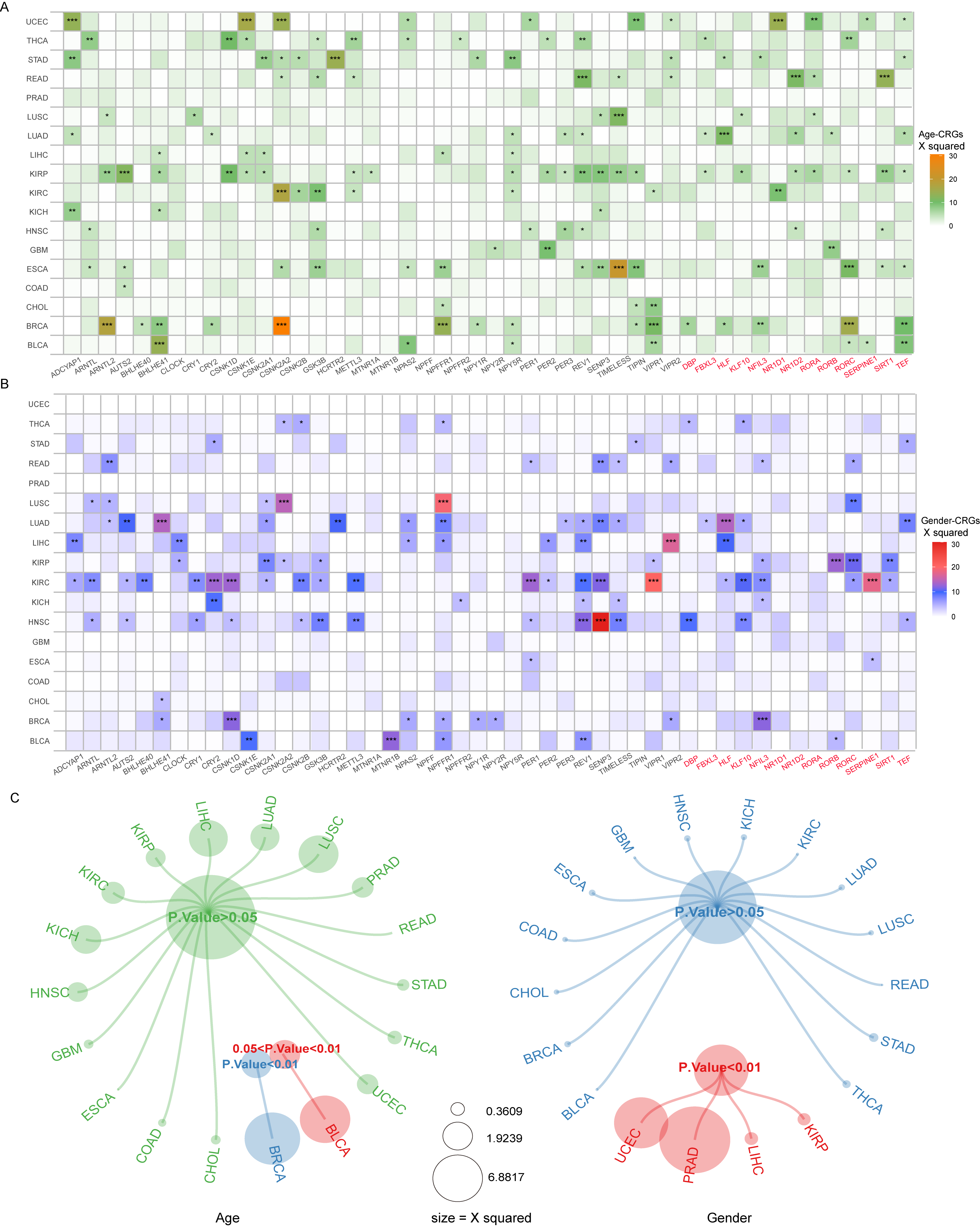

Supplement: Supplementary file 2 — Additional file 2: Figure S2. Correlation of circadian rhythm genes expression levels with clinical features in cancer. (A) Correlation of circadian rhythm genes expression levels with age: *p < 0.05; **p < 0.01; ***p < 0.001. (B) Correlation of circadian rhythm genes expression levels with gender. (C) Correlation of CRS with age (left) and sex (right). [file 12967_2023_4013_MOESM2_ESM.tif]

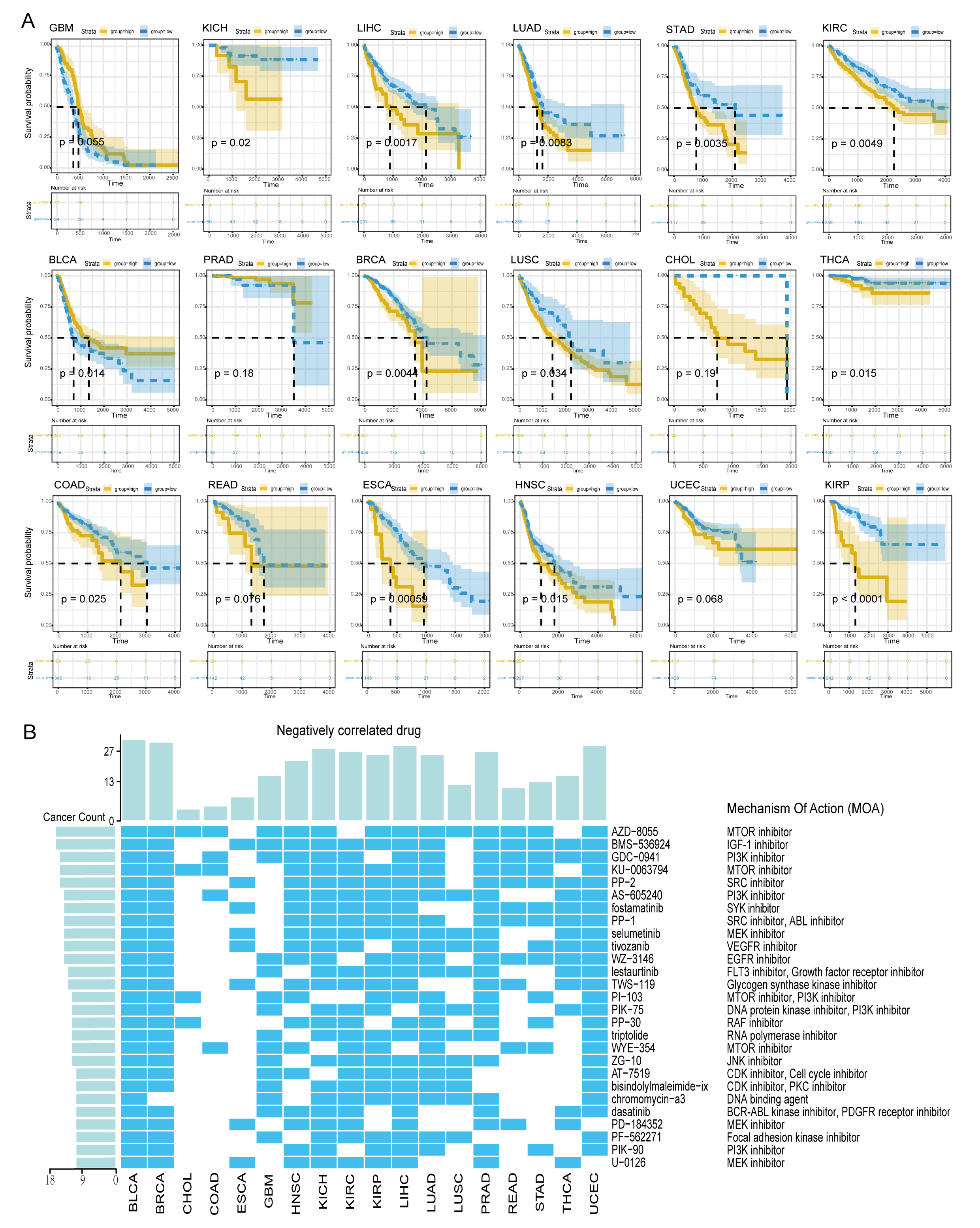

Supplement: Supplementary file 3 — Additional file 3: Figure S3. Comparison of CRS group survival and compounds negatively correlated with CRS. (A) Comparing the survival time of CRS subgroup samples by Log-rank method. (B) 18 compounds negatively correlated with CRS were identified by Cmap analysis. [file 12967_2023_4013_MOESM3_ESM.tif]
